# Phenotypic Plasticity and Selection: Nonexclusive Mechanisms of Adaptation

**DOI:** 10.1155/2016/7021701

**Published:** 2016-05-24

**Authors:** S. Grenier, P. Barre, I. Litrico

**Affiliations:** INRA, UR004, P3F, RD 150, Site du Chêne, BP 86006, 86600 Lusignan, France

## Abstract

Selection and plasticity are two mechanisms that allow the adaptation of a population to a changing environment. Interaction between these nonexclusive mechanisms must be considered if we are to understand population survival. This review discusses the ways in which plasticity and selection can interact, based on a review of the literature on selection and phenotypic plasticity in the evolution of populations. The link between selection and phenotypic plasticity is analysed at the level of the individual. Plasticity can affect an individual's response to selection and so may modify the end result of genetic diversity evolution at population level. Genetic diversity increases the ability of populations or communities to adapt to new environmental conditions. Adaptive plasticity increases individual fitness. However this effect must be viewed from the perspective of the costs of plasticity, although these are not easy to estimate. It is becoming necessary to engage in new experimental research to demonstrate the combined effects of selection and plasticity for adaptation and their consequences on the evolution of genetic diversity.

## 1. Introduction 

**Figure 1:**
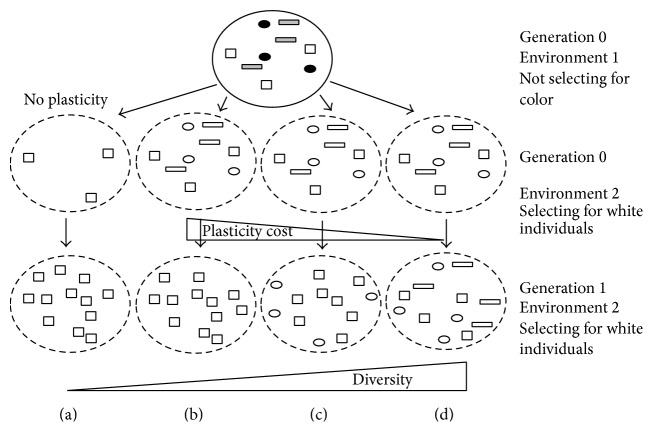
Impact of selection and plasticity on genetic diversity according to the cost of plasticity. Different forms represent genetic diversity and different colours represent different phenotypes and the population size is kept constant. (a) Without plasticity: strong decline of genetic diversity; (b) with equal plasticity among genotypes and a very strong cost of plasticity: strong decline of genetic diversity; (c) with equal plasticity among genotypes and a medium cost of plasticity for the circles and strong cost for the rectangles: low decline of genetic diversity; (d) with equal plasticity among genotypes and no cost of plasticity: maintenance of genetic diversity.

How do populations adapt to their environment? This is a crucial question in evolutionary biology and greater knowledge in this field is essential if we are to understand and preserve populations in a changing environment. Adaptation is the sum of the morphological, physiological, and behavioural changes of individuals that allows maintenance of individual fitness and so leads to population and species persistence. These changes arise as a result of two mechanisms operating at the level of the individual: selection and phenotypic plasticity. Under the first mechanism, genetic selection, the frequency of favourable alleles increases over several generations while that of unfavourable ones tends to decrease. The evolution of allele frequencies results from different reproductive efficiencies (fitness between genotypes) and so tends to reduce the genetic diversity within a population. The second mechanism, phenotypic plasticity, is defined as the ability of a genotype (individual) to express different phenotypes according to its environment [1]. A number of methods exist to quantify phenotypic plasticity with the use of various indices such as the trait mean, the trait variation coefficient, the trait reaction norm, and the trait extreme values and phenotypic distances [2]. Through phenotypic plasticity, the plastic traits of individuals are modified without modifying the genetic diversity of the population. But phenotypic plasticity can influence the fitness of individuals [3, 4] and be the target of selection [5–7]. The intensity of selection for plasticity depends on the balance between a positive effect and a negative one, the cost of plasticity on fitness. This balance can lead to different levels of genetic diversity within an adapted population [8–10] (Figure 1). Genetic diversity is essential in the long term, since it increases the ability of populations or communities to adapt to new environmental conditions [11–13]. However, despite the systematic coexistence of plasticity and selection in natural populations which encounter different environments (spatial and/or temporal changes) [14–16], there is a lack of experimental information on their combined influence on the evolution of the genetic diversity of populations. The purpose of this paper is to review current knowledge on the consequences of selection and phenotypic plasticity on the genetic diversity of populations and to propose procedures to overcome the limitations of experimental data in order to identify these consequences.

## 2. The Consequences of Selection on Genetic Diversity

Selection occurs on a trait only if it exhibits genetic variability within a population. Selection acts on different character types, physiological processes (e.g., resistance of plants to metals [21]), behaviours (e.g., predator avoidance [22, 23]), or morphological variations (e.g., industrial melanism of the peppered moth [24]). At the phenotypic level, selection changes the mean and/or the variance of trait distributions within a population. Selection can be of three types: directional, stabilising, or disruptive. Given a normal distribution of a trait, directional selection favours individuals with a phenotype either above or below the population mean and so leads to a shift of the mean in one direction or other [25]. Stabilising selection eliminates individuals with the most divergent phenotypes leading to a decrease in trait variance [26]. Disruptive selection favours individuals with extreme phenotypes, leading to a bimodal population distribution [27].

Response to selection results in allele frequency changes of genes involved in the phenotypic variation of traits implicated in individual fitness or at loci in linkage disequilibrium with the previous genes (hitchhiking). In addition to changes in allele frequencies of specific loci, selection can change the allele frequencies globally on the genome by genetic drift, due to a decrease in the effective size of the population. A strong selection can result in a rapid allele fixation (homogenisation of all individuals) and loss of other alleles. This can have a dramatic impact on the genetic diversity of a population (Figure 1). However, selection can also maintain alleles at intermediate frequencies through heterozygote advantage (individuals having two different alleles have greater fitness), or frequency dependence (the allele advantage depends on its frequency relative to other alleles in a given population).

Directional selection tends to decrease the genetic variability of a population in a given environment. But if several populations in different environments are considered (a metapopulation), the decrease in variability within each population can be compensated for by maintenance of diversity among populations [28–31]. Each environment selects for different alleles, allowing maintenance of all alleles in the metapopulation, even if some are lost at the individual population level. The principle of diversifying selection is used in genetic conservation studies.

## 3. The Consequences of Phenotypic Plasticity on Genetic Diversity

Phenotypic plasticity occurs at the individual level by changing the phenotype depending on the environment [10, 32–47]. For a given trait, this change with environment can be continuous or discrete [37]. In case of continuous phenotypic changes with an environment, the relationship between environmental variables and traits is called the reaction norms. These reaction norms are often complex and nonlinear. A plant or animal with a plastic trait receives a signal from the environment that will determine the trait value [36, 48–50]. The phenotypic changes are implemented during the lifespan of an individual; that is, the effects are seen within one generation; this is not the case for selection.

**Figure 2 fig2:**
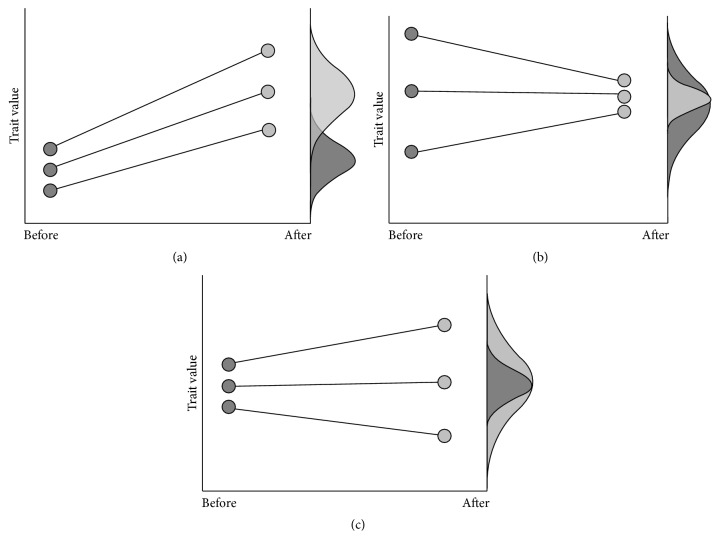
Scenarios for change in the mean and/or variance of a trait in a population between constitutive phenotypes expressed prior to an interaction (dark shading) and the induced phenotype following an interaction (light shading). (a) An increase in mean and variance of a trait. (b) A decrease in variance, mean unchanged. (c) An increase in variance, mean unchanged from [17].

**Figure 3:**
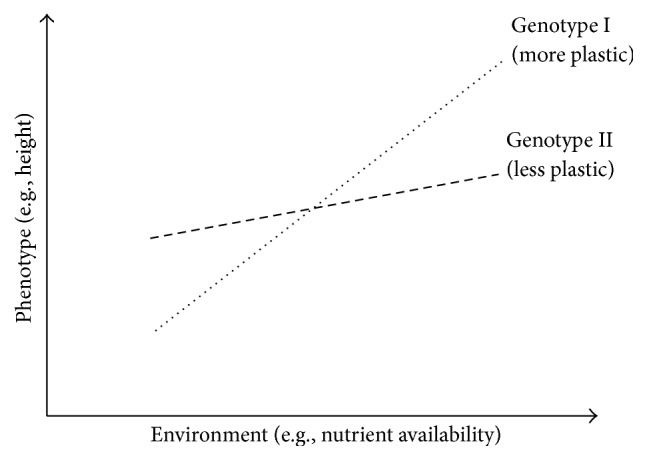
The lines represent the reaction norms (i.e. the genotype-specific environment-phenotype functions) of each genotype. Both lines have a slope in the environment-phenotype space, which means that both genotypes are plastic. The population shows GxE in the sense that there is genetic variation for the slope of the reaction norm, which would be detected by a standard analysis of variance from [18].

Phenotypic changes of individuals within a population will lead to a modification of the distribution of the phenotypic values of this population within one generation (Figure 2). This modification is reversible without any genetic changes but in some cases this modification is conserved over several generations. Therefore, if the shift of phenotypic values is beneficial to fitness (i.e., adaptive) this adaptation can be obtained without a decrease in genetic diversity (Figure 1 [Fig fig2]). In this case the phenotypic plasticity is adaptive and moderates the effects of selection. Some genotypes, not genetically adapted to an environment, become adapted through phenotypic plasticity and thus increase their fitness. Here it is necessary to introduce the notion of an* optimal phenotype* for a given environment. This is the phenotype which leads to the best fitness within the population. This optimal phenotype can be obtained with or without phenotypic plasticity. In a particular environment, if a genotype shows a phenotype close to the optimal phenotype, and if this phenotype is close to the average value of this genotype within a range of environments, it will have been obtained without phenotypic plasticity. In contrast, if another genotype shows a phenotype close to the optimal phenotype, and if this phenotype is far from being the average value of this genotype within a range of environments, then this phenotype will have been obtained through phenotypic plasticity. Within a population, the ability of phenotypic plasticity to moderate selection will depend on (i) the diversity of the traits involved in the optimal phenotype, (ii) the level of plasticity of the traits involved in the optimal phenotype, and (iii) the cost of the plasticity required to reach the optimal phenotype. This cost can be defined as the difference in fitness between the genotypes showing the optimal phenotype without plasticity and that of the genotypes displaying the optimal phenotype through plasticity. If the cost of plasticity is low enough, so that plastic genotypes have fitness as good as the best nonplastic genotypes, then the plastic genotypes will be selected for in the next generation. In such cases, with different levels of plasticity among genotypes (Figure 3), selection for plasticity will occur and will lead to a decrease in genetic diversity on the loci involved in plasticity variability. These can be different from the loci involved in the variability of the trait. Scheiner and Lyman [44] have shown in* Drosophila* that the plasticity of a trait can respond to selection and that this response is at least partially independent of the response of the trait mean. However, this independence remains controversial [51–53]. Within a population subject to different environments, adaptation over several generations comes from a balance between phenotypic plasticity and selection for both the value of adaptive traits and their plasticity. It is extremely rare to find studies that separate and quantify the roles: (1) of selection for the traits involved in the optimal phenotype, (2) of plasticity for these traits, and (3) of selection for plasticity of these traits. Nevertheless, each of these mechanisms has a different effect on the genetic diversity of the population.

## 4. Cost of plasticity

Phenotypic plasticity can act on fitness [54] which can become either closer to or further from the optimal phenotype [43]. Depending on the effect of plasticity on individual fitness, plasticity can be characterised by a number of terms. Phenotypic plasticity is termed as “maladaptive” when the effect on fitness is negative, “neutral” if it has no effect on fitness, and “adaptive” when it has a positive effect on fitness [35]. There are various examples of adaptive plastic responses. Thus, a plastic response has been shown in the case of a ciliate species following the introduction of a predator. The ciliate developed lateral wings which increased its survival by inhibiting ingestion by the predator. In turn, the predator responded plastically by increasing its gape size to accommodate the ciliate's defence without cancelling it (mutual plasticity) [3]. Some examples of adaptive plastic responses in plants are the lengthening of internodes in the shade to better access light [55], various herbivore-resistance mechanisms [56], or adjustments to the number of simultaneously mature flowers depending on the number of pollinators present [57]. In some cases, adaptive plasticity can become maladaptive if the environment becomes too stressful [43]. For example, when competition for light is very strong, some plants can increase their internode length but if the neighbouring plant species are too tall, the plastic capacity of increasing internode length for the smaller genotypes becomes maladaptive [58–60].

Even adaptive phenotypic plasticity could come at a cost [61, 62]. Lind and Johansson [63] suggest the cost increases with the level of plasticity in the population. This cost depends on genotype, population, and environment and can have quite a large number of origins [19]. These may include (i) maintenance costs (sensory structure, metabolic regulation mechanism of plasticity) [64], (ii) production costs (a plasticity-developed structure could be more costly than a genetically determined one) [65], (iii) acquisition of information costs (the process may be risky or it requires energy or reduces feeding or breeding efficiency), (iv) cost of imprecise development (nonspecific plastic change), (v) genetic costs (genetic linkage with deleterious alleles at other loci, pleiotropy, and epistasis) [66], and ecological costs (interaction between species; e.g., the plastic response to herbivory reduces attractiveness to pollinators) [67].

**Figure 4:**
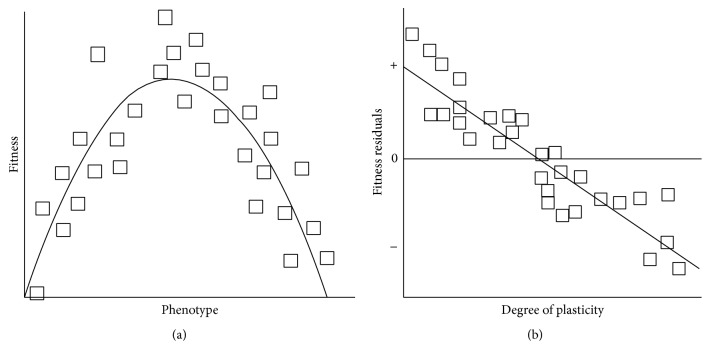
(a) Relationship between phenotypic values and fitness in one environment for population genotypes. (b) Relationship between degree of plasticity and fitness residuals. There is a cost of plasticity when the slope of the regression is negative from [19].

The costs of plasticity are difficult to quantify [68–70], but they can be estimated by comparing the fitness of plastic genotypes to that of fixed genotypes, for the same phenotypic value in each environment using the multiple regression: *W*_*j*_ = constant + (*B*_1_ × *Z*_*j*_)+(*B*_2_ × *P*_*j*_), where *W*_*j*_ is the fitness of family (*j*) in one environment, *B*_1_ is the selection coefficient on phenotypes, *B*_2_ is the selection coefficient on plasticity, *Z*_*j*_ is the phenotype exhibited by family *j* in one environment, and *P*_*j*_ is the plasticity exhibited by family *j* in different environments [71–73]. A significantly negative regression coefficient of the plasticity term indicates a genetic cost. This cost could be different in each environment. DeWitt et al. [19] adopt this method and analyse it to quantify the relationship between the phenotype and the fitness in each environment by regression analysis or by cubic splines [74]. This adaptation has the advantage of allowing nonlinear relationships between phenotype and fitness. This mathematical relationship allows calculation of a theoretical fitness that can be compared with the observed one. To visualise the trend, the residues of the fitness equation are shown graphically, based on the genotype degree of plasticity. There is a cost when the most plastic genotypes tend to have a lower fitness than expected and also when the fixed genotypes tend to have a higher fitness than expected (Figure 4). This method is interesting but a bias occurs when the phenotypic values are correlated with phenotypic plasticity [75]. This correlation must therefore be confirmed before calculating the cost of plasticity using this method. In several studies, the most extreme phenotypes have been associated with high plasticity [56, 73], which requires a reinterpretation of the results. If costs of plasticity are very high, they may have a considerable impact on the evolution of plasticity [14, 71, 76, 77]. Although as indicated by the analysis of Van Buskirk and Steiner [78] in both animals and plants, costs of plasticity are often low and can influence phenotype evolution under stressful conditions only. The ubiquity of plasticity suggests the associated benefits outweigh the costs under a wide range of conditions [10].

## 5. Selection, Plasticity, and Diversity: A Balance between Gain of Fitness and Cost

If the plastic response is close to the optimal phenotype, the cost of achieving the phenotype by plasticity will determine the impact of selection on the plastic individual and therefore on allelic diversity of loci involved in trait value. If the cost of plasticity is significant, then achieving the optimal phenotype via fixed development is better than doing so via phenotypic plasticity. Individuals having a mutation that achieves the optimal phenotype directly will be selected in a stable environment. There is a kind of genetic evolution that results in reducing plasticity and in reducing the diversity of a trait. Due to the cost of plasticity, the evolution of plasticity is slower than evolution due to maladaptive plasticity (see above) for which the selection intensity is generally stronger.

If the plastic response is still some way off from being the optimal phenotype but is still favourable, a genetic evolution by directional selection can complete it and bring it closer to the optimal phenotype. In this case, to obtain the phenotype, there is both a plastic response and a change related to selection [54].

However, depending on the conditions encountered by populations and their initial states, several hypotheses can be formulated for adaptive plasticity. Plasticity can, for example, moderate the effects of natural selection by allowing individuals to adapt phenotypically to new conditions quickly. Chevin et al. [79] have developed a model to predict population persistence in a changing environment. It can estimate how plasticity might interfere with the capacities of populations to evolve in response to environmental change.

By reducing the response to selection, phenotypic plasticity may promote the maintenance of genetic diversity within a population. If individuals have plastic traits, they can adjust their phenotypes to the environment. Different genotypes with several different phenotypes in one environment may have the same phenotype in another environment and therefore some genetic variability is maintained. Genetic variations that are few or are not expressed in an environment due to plasticity [80] become neutral if there is no cost of plasticity and thus they avoid a decrease in fitness. These predictions emerge from theoretical models [35, 81, 82] but there is little experimental data to verify them [8, 9]. In* Quercus coccifera*, a tree species with several ecotypes, Baquedano et al. [83] show that phenotype plasticity hides differences between genotypes which maintain a genetic variability that is inaccessible to selection.

However, we can find populations having low genetic diversity that are maintained with the help of plasticity. Plasticity can allow morphological adaptation without the need for genetic variability. The study by Geng et al. [46] compares two species in which morphological variance is small. The morphological variance in one of the species is due mainly to genotypic variations, while in the other species, which has low genetic diversity, the morphological variance is due to plasticity.

As plasticity influences genetic changes by affecting the intensity of selection, we can expect an overall change in genetic diversity through reorganisation of the evolutionary dynamics and thus of the evolution rate [84]. Intensity of selection varies with plasticity, thus upsetting the expected evolution in the absence of plasticity [17, 85, 86]. In a population of lizards (*Urosaurus ornatus*), a directional selection has been shown to favour the fastest-moving males. However, a seasonal plasticity increases the fitness of the slowest individuals during the breeding season. This seasonal plasticity homogenises the speed of the male population at breeding time and reduces the effect of selection for high speed [87].

**Figure 5:**
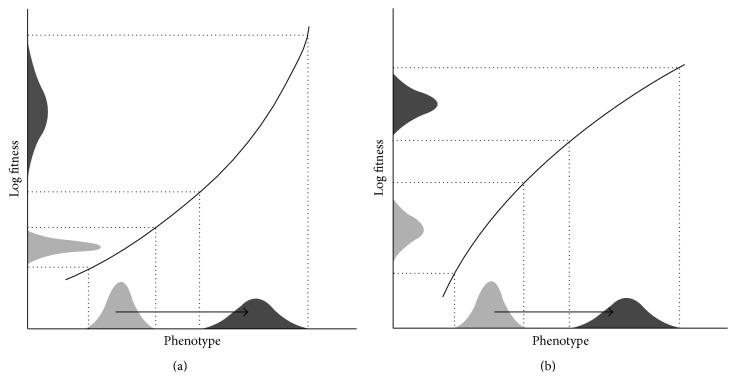
Schematic representation of the directional plasticity effects on the variation in the genotypic values *x*. The solid line shows the log of the fitness function, and the shaded areas symbolize the distributions of phenotypes and fitness without plasticity (light shading) and with plasticity (dark shading). Directional plasticity (symbolized by an arrow) shifts the distribution of the phenotypes (mean and variance). The curvature of the log fitness function (convex in (a); concave in (b)) determines how the shift in the mean of the distribution changes the variation in log fitness from [20].

To assess the impact of selection and plasticity on the evolution of genetic diversity, models allow us to assess the rate of the genetic changes caused by plasticity. Hinton and Nowlan [88] developed a model that shows how learning increases the rate of evolution. This model was modified by Behera and Nanjundiah [89] to obtain greater realism. They used a haploid population of fixed size, different levels of plasticity, and a cost associated with the plasticity. Their results indicate that plasticity slows down the pace of evolution but enhances the level of adaptation. However, other models of the same type, which explored more parameters, obtained mixed results [90–92]. To estimate the evolution rates, Ancel [93] used a quantitative model and a reaction norm model (modelling of plasticity by reaction norm instead of by genotype, phenotype points). Ancel's model is highly dependent on initial population conditions and on fitness. However, in most cases, the simulations obtained show that plasticity slows the evolution rate from the appearance, up to the stabilisation of the population at an optimal phenotype. When plasticity is not particularly costly, it protects suboptimal phenotypes from elimination by natural selection. In contrast, when plasticity is more costly, it fails to provide significant advantage. Furthermore, the phenotype ranges for which plasticity speeds up evolution are frequently those for which a plastic individual is less fit than its nonplastic counterpart. Paenke et al. [20] have sought conditions under which evolution is accelerated or delayed by plasticity under directional selection. According to these authors, the fitness gain gradient measures the effect of a marginal change in the degree of plasticity on the slope of the relationship between the genotypic value of the focal trait and log fitness. If the gain gradient has the same sign as the direction of selection, an increase in plasticity will magnify the response to selection. Conversely, if the two signs are opposite, greater plasticity will lead to a slower response. If plasticity acts similarly on genotypes, independently of their fitness, evolution is (i) accelerated if the logarithm of the fitness function (defined by the relationship between phenotype and fitness) follows a convex curve and (ii) slowed if this curve is concave (Figure 5). However, De Jong [94] considers the possibilities of evolution in the presence of plasticity and explains that there is no answer to the question of whether phenotypic plasticity promotes evolution. Everything depends on the case being studied and on the parameters associated with it.

## 6. Conclusion 

The study of phenotypic plasticity has developed rapidly in recent decades, so we now have a clearer view of what it is and how it evolves. But for the interaction of selection and plasticity, more experimental work is required to better understand how selection acts on the reaction norm of phenotype against environments and also to better define the costs of plasticity. It should be noted that most studies focus on two environments. There is a scarcity of data on environment ranges, such as those commonly experienced by natural populations and on the changes in the selection pressures generated. In addition, measuring plasticity is difficult because the global plasticity scale of the individual is relative to the plasticity of the different traits of the individual [95]. Efforts should be made to estimate the global effect of plasticity on fitness of several traits. These studies would be improved with the development of a better understanding of the mechanisms involved, especially with regard to the genetic basis that explain plasticity. Despite numerous theoretical studies, there remains a lack of experimental data to support the results of these studies. Indeed, it is difficult to obtain experimental data in natural ecosystems where it is almost impossible to follow generations, to have replicated genotypes, and to control the environment.* Ex situ* experimentation could circumvent these limits by using (i) species which can be cloned or selfed and thus are easy to raise/grow and phenotype such as plant species, (ii) populations showing genetic diversity of adaptive traits and their plasticity, (iii) species with short life cycles, and (iv) semicontrolled environments. In the context of climate change, it would be interesting to conduct studies on crops showing these characteristics to define ways which can be used to maintain production under increasingly hazardous climates events.
